# Animal-Assisted and Pet-Robot Interventions for Ameliorating Behavioral and Psychological Symptoms of Dementia: A Systematic Review and Meta-Analysis

**DOI:** 10.3390/biomedicines8060150

**Published:** 2020-06-02

**Authors:** Sangki Park, Ahream Bak, Sujin Kim, Yunkwon Nam, Hyeon soo Kim, Doo-Han Yoo, Minho Moon

**Affiliations:** 1 Department of Occupational Therapy, Konyang University, 158, Gwanjeodong-ro, Seo-gu, Daejeon 35365, Korea; sangki0222@gmail.com; 2 Department of Occupational Therapy, Jeonju Kijeon College, 267, Jeonjucheonseo-ro, Wansan-gu, Junju 54989, Korea; orang43@naver.com; 3 Department of Biochemistry, College of Medicine, Konyang University, 158, Gwanjeodong-ro, Seo-gu, Daejeon 35365, Korea; aktnfl3371@naver.com (S.K.); yunkwonnam@gmail.com (Y.N.); sooya1105@naver.com (H.s.K)

**Keywords:** dementia, behavioral and psychological symptoms of dementia, systematic review, meta-analysis, animal-assisted intervention, pet-robot intervention

## Abstract

Patients with dementia suffer from psychological symptoms such as depression, agitation, and aggression. One purpose of dementia intervention is to manage patients’ inappropriate behaviors and psychological symptoms while taking into consideration their quality of life (QOL). Animal-assisted intervention (AAI) and pet-robot intervention (PRI) are effective intervention strategies for older people with cognitive impairment and dementia. In addition, AAI and PRI have been shown to have positive effects on behavioral and psychological symptoms of dementia (BPSD). However, studies into the association between AAI/PRI and BPSD have elicited inconsistent results. Thus, we performed a meta-analysis to investigate this association. We analyzed nine randomized controlled trials on AAI and PRI for dementia patients published between January 2000 and August 2019 and evaluated the impact of AAI/PRI on agitation, depression, and QOL. We found that AAI and PRI significantly reduce depression in patients with dementia. Subsequent studies should investigate the impact of AAI and PRI on the physical ability and cognitive function of dementia patients and conduct a follow-up to investigate their effects on the rate of progression and reduction of symptoms of dementia. Our research will help with neuropsychological and environmental intervention to delay or improve the development and progression of BPSD.

## 1. Introduction

In 2016, it was estimated that 47 million individuals are living with dementia worldwide, and this figure is projected to increase to 113 million in 30 years. As a result, the public health burden of dementia is anticipated to significantly increase in the coming years [1]. Currently, the World Health Organization is striving to promote dementia prevention and increase dementia awareness by significantly investing in health and welfare and active research into dementia [2]. Furthermore, many countries have implemented national strategies aimed at optimizing dementia management in preparation for the anticipated burden of dementia and its effects on their healthcare system [3]. Dementia patients commonly suffer from behavioral and psychological symptoms of dementia (BPSD) [4]. BPSD include socially inappropriate neurobehavioral symptoms such as mental and emotional symptoms, hyperactivity, and sleep disorders [5]. Depression and agitation are the most common emotional problems that affect dementia patients [4]. The goal of dementia treatment is to manage patients’ inappropriate behaviors and psychological symptoms while considering their quality of life (QOL), and active research into cognitive stimulation therapy, a nonpharmacological intervention for dementia, is ongoing [6]. However, previous studies into therapies for dementia have generally focused on their effect on cognitive abilities such as memory, problem-solving ability, and communication skills, and the impact of these therapies on the psychological and social aspects of dementia has been neglected [7]. Recently, many interventions for the treatment of BPSD have received attention [8,9,10], including animal-assisted interventions [11,12].

Animal-assisted interventions (AAI) are interventions that involve animals. There are various subgroups of AAI, namely animal-assisted activities (AAA), animal-assisted therapies (AAT), and service animal programs (SAP) [13]. These are known to be effective interventions for older people with cognitive impairment, and recent studies have reported that AAI have positive effects on dementia patients [14]. AAA refer to unofficial activities involving animals that meet certain requirements and are characterized by a certain level of flexibility and spontaneity. AAT refer to interventions involving animals that are aimed at improving certain patient outcomes and are incorporated into rehabilitation programs [15]. SAP refers to programs that utilize trained animals to help clients with physical disabilities to overcome functional difficulties in their activities of daily living [16]. These interventions provide joy to patients, increase their motivation, and allow them to rest [17], and patients are able to resolve their unmet physical and emotional needs by being involved in activities related to patients therapeutic goals [18]. In particular, walking a living animal is not only beneficial to dementia patients but also facilitates the rehabilitation of adults who have undergone surgery or have an illness by reacquainting them with ambulation and recovering ambulation speed [19,20,21]. The first AAI to be developed were found to reduce depression [22], and the ability of AAI to reduce depression and improve QOL in older people with dementia is currently being investigated [11,23,24,25]. Despite the known benefits of AAI, their use is restricted in some medical environments due to concerns about patients having a fear of animals, possible infection risk, and fright [26].

Recently, pet-robot intervention (PRI) has been proposed as an alternative to AAI. PARO, the most widely studied PRI, is a seal-shaped robot which responds to light, temperature, touch, and posture and monitors the client’s emotional changes and health status using sensors [27]. PARO is reported to have various beneficial psychological and social effects such as promoting interaction, reducing stress, and alleviating depression. Furthermore, PRI has similar effects to AAI involving living animals; overcomes the limitations associated with living animals; and has cost, hygiene, and safety benefits [28]. Notably, one study reported that PARO has a positive impact on depression and psychological agitation in older people with dementia and concluded that PARO is a nonpharmacological intervention effective at alleviating neuropsychiatric symptoms [29]. Furthermore, PARO alleviated stress and agitation and reduced the use of antipsychotics and analgesics in older people with dementia [30].

It is well-known that AAI and PRI have beneficial effects on symptoms of dementia [23,30]. In addition, systematic reviews of the effect of AAI or PRI on symptoms of dementia have been performed [31,32,33]. However, no studies have been conducted into the effects of both AAI and PRI on BPSD. Therefore, the aim of this systemic review and meta-analysis was to investigate the effects of AAI and PRI on BPSD and to present clinical evidence for the application of these interventions.

## 2. Results

### 2.1. Characteristics of the Included Studies

Nine studies met the inclusion criteria for this study, and their general characteristics are presented in Table 1. Only studies with a PEDro score of 4–7 and thus deemed to be of “fair” or “good” quality were included [34]. A total of 507 participants were included in the meta-analysis. In the included studies, dementia patients were subjected to various interventions involving living or robotic animals. Each study was systematically analyzed and compared with the rest of the studies. The control group was typically subjected to the conventional treatment program provided at the hospital or facility at which the study was conducted.

### 2.2. Meta-Analysis of the Effects of AAI and PRI

#### 2.2.1. Meta-Analysis of the Effects of AAI and PRI on Agitation in Dementia Patients

**Figure 1:**
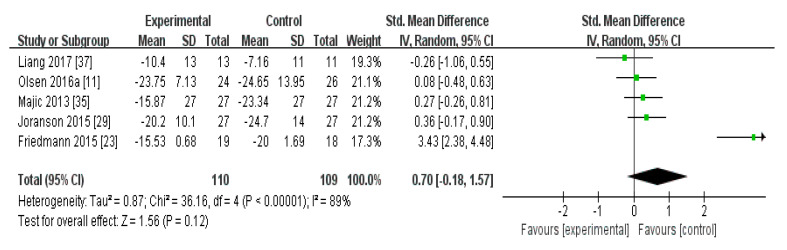
Forest plot of the effect of animal-assisted intervention and pet-robot intervention on agitation in dementia patients.

In the meta-analysis of the effects of AAI and PRI on agitation in dementia patients, the effect size was 0.70 (95% confidence interval: *p* = 0.12, I^2^ = 89%), which was considered a large effect size. Overall, AAI and PRI did not significantly affect agitation in dementia patients (Figure 1).

#### 2.2.2. Meta-Analysis of the Effects of AAI and PRI on Depression in Dementia Patients

**Figure 2:**
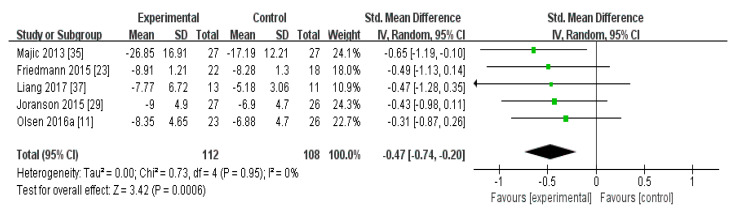
Forest plot of the effect of animal-assisted intervention and pet-robot intervention on depression in dementia patient.

In the meta-analysis of the effects of AAI and PRI on depression in dementia patients, the effect size was −0.47 (95% confidence interval: *p* < 0.001, I2 = 0%). Overall, AAI and PRI significantly reduced depression in dementia patients (Figure 2).

#### 2.2.3. Meta-Analysis of the Effects of AAI and PRI on the QOL of Dementia Patients

**Figure 3:**
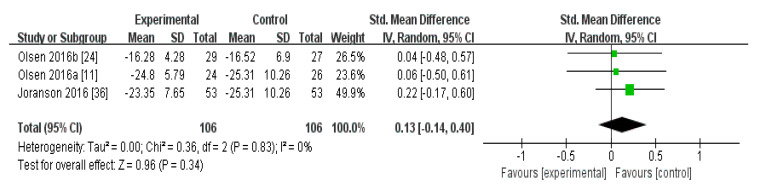
Forest plot of the effect of animal-assisted intervention and pet-robot intervention on the quality of life of dementia patients.

In the meta-analysis of the effects of AAI and PRI on the QOL of dementia patients, the effect size was 0.13 (95% confidence interval: *p* = 0.34, I^2^ = 0%), which was considered a small effect size. Overall, AAI and PRI did not significantly affect the QOL of dementia patients (Figure 3).

#### 2.2.4. Publication Bias

**Figure 4:**
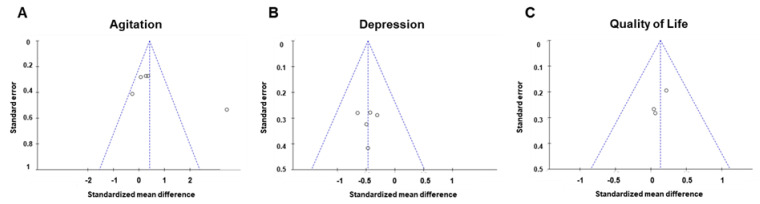
Funnel plots used to assess the existence of publication bias in the included studies. Publication bias of (**A**) agitation, (**B**) depression, and (**C**) quality of life.

When publication bias with respect to agitation, four studies were within the 95% confidence interval and were plotted to the left of the overall effect estimate (Figure 4A). When publication bias with respect to depression and QOL with respect to the effect of AAI and PRI was assessed (Figure 4B,C), all plotted dots were within the 95% confidence interval.

## 3. Discussion

Currently, more than 90% of dementia patients suffer from BPSD [39], which poses major difficulties to both dementia patients and their caregivers. The type of BPSD varies according to dementia type, stage of the illness and various other factors. Particularly, patients of frontotemporal lobar degeneration (FTLD) show more prominent behavioral variants such as disinhibition, impulsivity, aggression, and personality change than those with other types of dementia [40,41,42]. Another study demonstrated that patients with dementia with Lewy bodies (DLB) present hallucinations and aberrant motor behavior (AMB) more so than Alzheimer’s disease (AD) patients [43,44]. An increased rate of anxiety, depression, and psychosis may occur in vascular dementia (VD) [40,43,45]. Depression and agitation are the most common symptoms affecting various dementia patients. Furthermore, it is known that agitation, apathy, disinhibition, irritability, and motor dysfunction become serious as dementia progresses. In particular, depression and anxiety become more severe in the moderate stage of dementia [46,47,48]. In the early stages of dementia, apathy mainly appears, which is one of the first symptoms of the various forms of dementia. Apathy is a dangerous barrier that affects social interaction and activities of daily living due to lack of interest, enthusiasm, and apathetic response to interpersonal communication [49]. These psychological and behavioral changes from the early stages of dementia can affect aspects of BPSD such as depression and anxiety more seriously as dementia progress. Although BPSD, which varies depending on the type and progression of dementia, contains a range of important symptoms that affect the quality of life, stress, and prognosis of dementia patients and their caregivers, there is little of interest in and study on nonpharmacological interventions to treat BPSD. Thus, we performed a meta-analysis to investigate the effect of AAI and PRI—one of the nonpharmacological interventions using animals— on agitation, depression, and QOL in dementia patients [15,26,27].

The meta-analysis of the effects of AAI and PRI on agitation showed a medium effect size of 0.70 (Figure 1). Three studies that utilized AAI and two studies that utilized PRI were included in the meta-analysis. The studies that used AAI reported larger effect sizes than those that used PRI, but AAI and PRI were not found to significantly affect agitation overall [23,24,35]. Our result contrasts with the results of a previous study which showed an alleviation in the agitation. However, since the level of evidence for the randomized controlled trials (RCTs) in previous studies was very low, we thought that the opposite results were obtained. Accordingly, our results support the suggestion of previous studies that the level of evidence is low [32].

The meta-analysis of the effects of AAI and PRI on depression showed a medium effect size of −0.47 (Figure 2). Three studies that used AAI were included, and two reported that this intervention strategy reduced depression [23,24,35]. Two studies that used PRI were included, and these showed a medium effect size [36,37]. AAI and PRI were found to significantly reduce depression, which serves as evidence that AAI and PRI are effective at reducing depression in dementia patients (*p* < 0.001).

The meta-analysis of the effects of AAI and PRI on QOL showed a small effect size of 0.13, but the results were not statistically significant (*p* > 0.05) (Figure 3). Two studies used AAI, and both reported that these interventions improved QOL [11,24]. One study used PRI, and reported that this intervention did not significantly affect QOL [36]. The meta-analysis results showed that AAI and PRI did not significantly affect QOL, which supports previous findings [32].

The present study analyzed the effects of AAI and PRI on BPSD and found that these interventions did not affect agitation or QOL but significantly reduced depression. It is well known that the brain with depression in dementia has reduced connectivity on amygdala and emotion control regions [50,51]. AAI and PRI provide an emotional effect and a and sense of closeness to dementia patients [52], which may the reduced amygdala connectivity in dementia patients. In addition, AAI and PRI could have a positive effect on hippocampus in the brain with depression through activities that require memory, such as checking the health of animals, walking, and feeding. On the other hand, the agitation-related connectivity is the orbital frontal cortex and anterior cingulate cortex, which is a region that has little association with emotional support obtained through activities with animals. Thus, AAI and PRI did not show a significant effect in agitation. Although AAI and PRI have been effective in improving depression, it is difficult to dramatically relieve all BPSD symptoms. Moreover, it is known that BPSD is specifically related with the patient’s low of QOL [53]. Therefore, in this study, it is considered that AAI and PRI were difficult to significantly influence QOL. A previous meta-analysis reported that AAI do not affect activities of daily living, depression, agitation, QOL, or cognitive function. In addition, a number of limitations are associated with interventions involving the use of living animals: patients may be fearful of or allergic to animals, animals may provoke falls in vulnerable patients, and animals may pose an infection risk to patients [32]. Moreover, there are a number of difficulties associated with managing animals—they need to be fed, produce feces, and may smell. However, it is clear that AAI can enhance the emotional wellbeing and QOL of dementia patients. Although robotic animals cannot evoke the same variety of emotions and sensations as living animals, they are easier to manage and could aid patients wherever needed. Subsequent studies should additionally examine the impact of living animals and robotic animals on the emotional wellbeing, cognitive function, and physical ability of dementia patients. Furthermore, patients should be followed-up to investigate the efficacy of these interventions in slowing the progression of dementia.

Several studies have suggested that psychiatric symptoms such as depression and anxiety are associated with dementia and cognitive impairment [54,55,56]. Indeed, patients with dementia have an increased risk of major depression, and many suffer from anxiety [57,58]. Interestingly, amyloid-beta (Aβ) burden and tau-related pathology are known to worsen in Alzheimer-type dementia with depression [55,59]. In addition, depression and agitation are causative factors of sleep disorders, and they can promote the development of dementia by inhibiting Aβ clearance and inducing systemic inflammation [60,61,62,63]. Therefore, it is important to alleviate the psychological symptoms of dementia patients. In this study, we confirmed that AAI and PRI can relieve the psychological symptoms of dementia patients. Several mechanisms by which AAI and PRI may affect BPSD have been proposed. First, AAI and PRI affect hormone levels. Previous studies consistently reported that dog-raising people exhibit higher levels of oxytocin, a hypothalamic neuropeptide [64,65]. Oxytocin is closely related to cognitive function, depression, agitation, and social communication and has been proposed as a pharmacological intervention for neurobehavioral disorders in patients with prefrontal dementia [66,67]. In addition, it has been reported that animal owners exhibit reduced cortisol levels [68]. In AD, cortisol levels substantially increase and this steroid hormone elicits neurotoxic effects in the hippocampus and thus exacerbates Aβ pathology and contributes to cognitive impairment [69]. Therefore, AAI may improve BPSD by increasing oxytocin levels and reducing cortisol levels. Furthermore, the relationship between loneliness and depression is well established, and loneliness has been reported to promote Aβ deposition in the brain of AD patients [70,71]. In addition, loneliness is known to contribute to cognitive decline by lowering cognitive reserve [72]. Surprisingly, AAI is known to reduce the loneliness of residents in long-term care facilities [73]. Therefore, AAI and PRI may effectively reduce loneliness and depression in dementia patients.

Second, it is possible that AAI and PRI modulate brain structure and functional connectivity. Patients with dementia exhibit atrophy of the hippocampus and entorhinal cortex, areas of the brain associated with emotional and spatial memory [74]. In addition, late-stage dementia is associated with dysfunction of the amygdala and cerebral cortex [75,76]. Accordingly, patients with dementia have problems with language, reasoning, emotions, and social behavior. Furthermore, atrophy of the hippocampus and cerebral cortex affects the functional connectivity of frontotemporal and limbic circuits involved in depression and mood regulation [77]. Strikingly, emotion-related brain areas may be affected by dementia patients’ relationship and emotional stability. Indeed, improvements in executive function, social skills, mood regulation, learning, memory, and attention were noted in patients receiving cognitive rehabilitation therapy through various AAI [52]. In addition, in children with ADHD, AAI had a calming effect, increased motivation, improved cognitive function, and promoted socialization [78]. It is thought that interaction with a therapy animal enhances functional connectivity between the frontotemporal and limbic systems. Moreover, having to look after an animal and remember to perform tasks such as feeding it is thought to improve memory and learning ability and attenuate hippocampal and cortical atrophy. Social interaction is possible through relationships and walking with animals, and through group meetings, depression will be alleviated. Although the neurological mechanisms underlying the effects of AAI and PRI have not been fully elucidated, accumulating evidence suggests that AAI and PRI can effectively improve BPSD.

Although a number of previous studies have also investigated living- and robotic- animal-assisted interventions for patients with dementia, our study has a number of strengths [31,32,33]. First, we comprehensively investigated the effects of interventions involving living and robotic animals and, for the first time, compared the effects of AAI and PRI on BPSD. Second, we demonstrated trends in research in this field and confirmed that more research is now being conducted into interventions involving robotic animals for dementia patients. Third, two reviewers independently identified articles that met the inclusion criteria, and a high level of inter-rater agreement was noted. Fourth, we focused on BPSD and dementia. Although AAI and PRI are known to affect various symptoms of dementia patients, we conducted a literature search and meta-analysis focusing on BPSD. Finally, it is difficult to distinguish between mild cognitive impairment (MCI) and dementia patients unless a neurological examination is performed to definitively diagnose dementia. In this study, we aimed to confirm the effect of AAI and PRI in individuals who had been diagnosed with dementia, not MCI.

Nevertheless, our study has a number of limitations. One limitation of the meta-analysis is the small number of included studies, which shows that there is a lack of literature relating to AAI and PRI for dementia patients. In addition, we only selected studies published in peer-reviewed journals and did not include any grey literature, which may have introduced publication bias. Third, we were unable to identify specific subgroups of dementia patients who may benefit most from AAI and PRI. Finally, we searched only a few English language databases, so some relevant studies may have been missed.

## 4. Methods 

### 4.1. Subsection

A meta-analysis was performed to analyze and validate studies that investigated the effects of AAI and PRI on dementia patients.

### 4.2. Search Strategy

**Figure 5:**
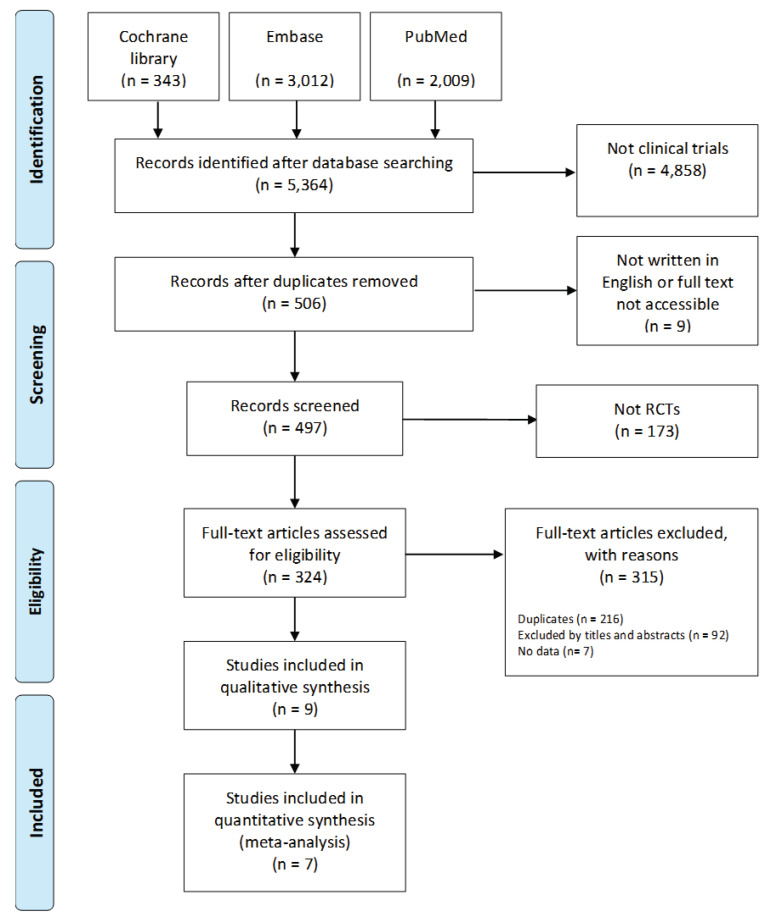
Flow chart of the systematic literature review.

Studies into the effect of AAI and PRI on dementia patients published between January 2000 and August 2019 were analyzed. Data were collected from three electronic databases—the Cochrane Library, Embase, and PubMed (Figure 5). The search terms used were “Dementia” AND “animal-assisted therapy OR animal-assisted activity OR service animal programs OR animal OR robot”. A total of 5364 studies were initially identified, and, after the exclusion of 4858 nonclinical trials, 506 studies underwent further analysis. An additional 506 studies were then excluded: 1 because the original text was unavailable, 9 because they were written in a language other than English, 173 because they were not RCTs, 216 because they were duplicates, 92 because they were inappropriate for the purpose of our study/because they were unsuitable based on a review of their titles and abstracts, and 7 because data were missing or disorganized. Ultimately, nine studies were included in the systematic review and meta-analysis.

### 4.3. Selection Criteria

Studies were included if they met all of the following criteria: (i) the study population comprised dementia patients, (ii) the experimental intervention was an AAI or PRI, (iii) the participants were randomized into groups, (iv) standardized evaluations were conducted to compare the effects of the intervention and control treatment, and (v) sufficient data were available to compute the effect size.

### 4.4. Study Selection and Data Extraction

Two reviewers (S.P. and A.B.) independently identified studies that met the inclusion criteria and performed data extraction. Disagreements between the reviewers were resolved by discussion. From each selected study, the following data were extracted: author, year of publication, mean age of the participants, study design (sample size, intervention type, follow-up duration, and frequency of intervention), and outcome measurement tools.

### 4.5. Qualitative Assessment of Study Methodology

One reviewer (S.P.) assessed the quality of the nine selected studies by assigning each a PEDro score (OTseeker, 2003), and the results were verified by the other reviewer (A.B.). The PEDro score ranges from 0–10 and the quality of a study is classified as “poor” (≤3), “fair” (4–5), “good” (6–8), or “excellent” (9–10) [34]. Studies deemed to be of “fair” to “good” quality (4–7) were included in this analysis. Any disagreements between the investigators with respect to the qualitative assessment of the studies were resolved by discussion.

### 4.6. Qualitative Assessment of Study Methodology

For each of the included studies, the following data were presented: name of first author/names of all authors, year of publication, age of participants, sample size, type of intervention/intervention method, duration and frequency of intervention, instruments used to assess primary outcomes, and PEDro score. To analyze the effects of AAI on dementia patients based on these characteristics, the mean, standard deviation, and sample size of the intervention and control groups were computed (Table 1). We examined whether the direction of the effect size was identical across studies and if not, made them equal by multiplying the mean by −1 [79].

### 4.7. Statistical Analysis

It is inappropriate to determine whether a fixed effect model or random effect model should be employed using the heterogeneity statistic I^2^. In order to select an appropriate effect model, the characteristics of the study, the subjects of the study, the method of intervention, and the mean value of the intervention effect were examined. In order to select an appropriate model to determine statistical heterogeneity, the characteristics of individual studies, study design, study subjects, intervention methods, and average values of intervention effects were examined [80].

Effect sizes were calculated to determine and compare the effect of AAI and PRI/different interventions on activities of daily living, stress, depression, and mental health using the sample size, mean, standard deviation, and statistically significant test of the experimental and control groups. According to the analysis criteria suggested by Cohen [81], 0.2 or less was considered a small effect size, 0.5 a medium effect size, and 0.8 or more a large effect size. The quantitative results of the meta-analysis were presented using forest plots. Publication bias was assessed by creating funnel plots. These were assessed by two reviewers and any disagreements were resolved by discussion. The chi-squared test was performed to determine the significance of the Q statistic [82,83]. If the *p*-value of Q was less than 0.10, there was deemed to be significant statistical heterogeneity between studies. A higher significance level was used since the Q statistic has low statistical power when only a small number of studies are included in a meta-analysis [84]. All statistical analyses were performed using Review Manager 5.3 software (RevMan; the Cochrane Collaboration, Oxford, UK).

## 5. Conclusions

This study systematically reviewed, compared, and meta-analyzed the impact of AAI and PRI on agitation, depression, and QOL in dementia patients. Interventions involving both living and robotic animals were investigated. The meta-analysis revealed that AAI and PRI interventions significantly reduced depression but did not affect agitation or QOL. Comparison of AAI and PRI showed that each method has its benefits and shortcomings and indicated that the two methods could potentially complement each other. Interventions involving living animals had a more beneficial effect on the emotional wellbeing of dementia patients than PRI. Although robotic animals overcome some limitations of living animals, they were not shown to alleviate BPSD in this study. In the future, more research should be conducted on the impact of living and robotic animals on the emotional wellbeing, cognitive function, and physical ability of dementia patients. Furthermore, we hope that AAI and PRI, which have been found to effectively reduce depression in dementia patients based on follow-ups, are more commonly utilized in clinical practice.

## Figures and Tables

**Table 1:** Characteristics of the included studies.

	Study	Participants (Experimental/Control)		Intervention					Outcome Measures	Pedro Score
		Age	Sample Size	Experimental Group		Control Group		Period/Total Number of Sessions		
1	Majic (2013) [35]	81.33 ± 10.20 /82.07 ± 8.65	27/27	AAI (Dog)	Dog-assisted intervention	Same care and treatment as before the study		10 weeks /10 sessions	MMSE, CMAI, DMAS *	4
2	Friedmann (2015) [23]	79.59 ± 9.74 /82.11 ± 8.36	22/18	AAI (Dog)	Dog-assisted intervention	Social skills and fine motor skills		12 weeks /24 sessions	AES, CSDD, CMAI	6
3	Olsen (2016a) [11]	65 or older	25/26	AAI (Dog)	Petting the dog, feeding the dog a treat, and throwing a toy for the dog to fetch	Music therapy, sensory garden, singing, exercise, cooking, and handicrafts		12 weeks /24 sessions	BARS, CSDD *, QUALID	6
4	Olsen (2016b) [24]	65 or older	22/26	AAI (Dog)	Petting the dog, feeding the dog a treat, and throwing a toy for the dog to fetch	Usual treatment		12 weeks /24 sessions	BBS, QUALID	5
5	Joranson (2015) [29]	83.9 ± 7.2 /84.1 ± 6.7	27/26	PRI (PARO)	Petting, talking to and about, smiling to, and singing to the robotic animal	Usual treatment		12 weeks /24 sessions	BARS *, CSDD *	6
6	Joranson (2016) [36]	83.9 ± 7.2 /84.1 ± 6.7	27/26	PRI (PARO)	Petting, talking to and about, smiling to, and singing to the robotic animal	Usual treatment		12 weeks /24 sessions	CDR, QUALID	7
7	Petersen (2016) [30]	83.5 ± 5.8 /83.3 ± 6.0	35/26	PRI (PARO)	Interaction activity of 6 people one group to PARO	Music therapy, physical activity, and mental stimulation		12 weeks /36 sessions	RAID **, CSDD **, GDS, pulse oximetry **, pulse rate **, GSR	6
8	Liang (2017) [37]	67–98	13/11	PRI (PARO)	Separate PAROs were provided to each participant’s home environment	Standard activities (quizzes, exercise, bingo, music, and word activities)		6 weeks /12–18 sessions	CMAI-SF, cognitive score, NPI-Q, depressive symptoms *	6
9	Moyle (2018) [38]	84 ± 8.8 /86 ± 7.6 /85 ± 6.9	67/55/53	PRI (PARO)	Participants were left alone with PARO for 15 min to interact with it as they liked	PARO with all artificial intelligence disabled	Usual treatment	10 weeks /30 sessions	Sense Wear *	7

AAI, animal-assisted intervention; AES, Apathy Evaluation Scale; BARS, Brief Agitation Rating Scale; BBS, Berg Balance Scale; CDR, Clinical Dementia Rating Scale; CMAI, Cohen-Mansfield Agitation Inventory; CMAI-SF, Cohen-Mansfield Agitation Inventory Short Form; CSDD, Cornell Scale for Depression in Dementia; DMAS, Dementia Mood Assessment Scale; GDS, Global Deterioration Scale; GSR, galvanic skin response; MMSE, mini mental state examination; PRI, pet-robot intervention; RAID, Rating for Anxiety in Dementia; QUALID, Quality of Life in Late-Stage Dementia Scale; NPI-Q, Neuropsychiatric Inventory Brief Questionnaire; * *p* < 0.05, ** *p* < 0.01.

## Abbreviations

AMB	aberrant motor behavior
AD	Alzheimer’s disease
Aβ	amyloid-β
AAA	animal-assisted activities
AAI	Animal-assisted intervention
AAT	animal-assisted therapies
BPSD	behavioral and psychological symptoms of dementia
DLB	dementia with Lewy bodies
FTLD	frontotemporal lobar degeneration
MCI	mild cognitive impairment
PRI	pet-robot intervention
QOL	quality of life
RCTs	randomized controlled trials
RevMan	Review Manager
SAP	service animal programs
VD	vascular dementia
